# Whole-genome mapping identified novel “QTL hotspots regions” for seed storability in soybean (*Glycine max* L.)

**DOI:** 10.1186/s12864-019-5897-5

**Published:** 2019-06-17

**Authors:** Xi Zhang, Aiman Hina, Shiyu Song, Jiejie Kong, Javaid Akhter Bhat, Tuanjie Zhao

**Affiliations:** Soybean Research Institution, National Center for Soybean Improvement, Key Laboratory of Biology and Genetics and Breeding for Soybean, Ministry of Agriculture, State Key Laboratory of Crop Genetics and Germplasm Enhancement, Nanjing Agricultural University, Nanjing, 210095 China

**Keywords:** QTL, Seed storability, High-density linkage map, Seed aging, Soybean

## Abstract

**Background:**

Seed aging in soybean is a serious challenge for agronomic production and germplasm preservation. However, its genetic basis remains largely unclear in soybean. Unraveling the genetic mechanism involved in seed aging, and enhancing seed storability is an imperative goal for soybean breeding. The aim of this study is to identify quantitative trait loci (QTLs) using high-density genetic linkage maps of soybean for seed storability. In this regard, two recombinant inbred line (RIL) populations derived from Zhengyanghuangdou × Meng 8206 (ZM6) and Linhefenqingdou × Meng 8206 (LM6) crosses were evaluated for three seed-germination related traits viz., germination rate (GR), normal seedling length (SL) and normal seedling fresh weight (FW) under natural and artificial aging conditions to map QTLs for seed storability.

**Results:**

A total of 34 QTLs, including 13 QTLs for GR, 11 QTLs for SL and 10 QTLs for FW, were identified on 11 chromosomes with the phenotypic variation ranged from 7.30 to 23.16% under both aging conditions. All these QTLs were novel, and 21 of these QTLs were clustered in five QTL-rich regions on four different chromosomes viz., Chr3, Chr5, Chr17 &Chr18, among them the highest concentration of seven and six QTLs were found in “QTL hotspot A” (Chr17) and “QTL hotspot B” (Chr5), respectively. Furthermore, QTLs within all the five QTL clusters are linked to at least two studied traits, which is also supported by highly significant correlation between the three germination-related traits. QTLs for seed-germination related traits in “QTL hotspot B” were found in both RIL populations and aging conditions, and also QTLs underlying “QTL hotspot A” are identified in both RIL populations under artificial aging condition. These are the stable genomic regions governing the inheritance of seed storability in soybean, and will be the main focus for soybean breeders.

**Conclusion:**

This study uncovers the genetic basis of seed storability in soybean. The newly identified QTLs provides valuable information, and will be main targets for fine mapping, candidate gene identification and marker-assisted breeding. Hence, the present study is the first report for the comprehensive and detailed investigation of genetic architecture of seed storability in soybean.

**Electronic supplementary material:**

The online version of this article (10.1186/s12864-019-5897-5) contains supplementary material, which is available to authorized users.

## Background

Soybean is one of the most important oil crop species for food, feed and of many industrial applications. This legume crop has originated in East Asia, and is now widely grown as primary oilseed crop with the United States, Brazil, Argentina, India and China are the major soybean-growing countries [1, 2]. In China, soybean production depends on maize-soybean intercropping, and the area under soybean crop has considerably increased in recent years [3, 4]. However, it is interesting that despite of this, China is currently the major soybean-importing country in the world for meeting the increasing requirement of plant protein, oil and food. Hence, it is an immediate need to improve soybean production for meeting the demand of its growing population.

Seed germination is a most important stage in the life cycle of plant, and it determines the distribution of wild species as well as the increased yield and quality of cultivated crop species [5, 6]. Generally, seed germination is completed following the emergence of the radicle [7]. Immediately after seed germination, another crucial development stage is seedling establishment in which transition occurs from heterotrophic to autotrophic state [8]. Therefore, for normal plant development both seed germination and seedling establishment are important. It is very important to mention that both these processes draw their energy from the seed stored in it [9]. However, after the seed is mature, it enters into the storage or dormancy phase. In crop species, as the time of storage for seeds increases, the irreversible process of declining seed vigor is initiated, called seed deterioration/aging [10]. Seed aging is an inevitable phenomenon in which the seeds lose their vigor and viability during the process of storage [11].

Seed storability is defined as the longevity of seeds after storage, and is important agronomic factor for the preservation of seed fitness after harvest [12]. Poor longevity causes loss of seed viability during storage, and negatively impacts seedling establishment and crop productivity [13, 14]. This is major problem for soybean seeds, which lost their vigor and viability rapidly during storage, especially in high temperature and humid environment [15]. By considering the relative storability index, soybean is categorized in the least storable group [16]. Many previous studies have revealed that soybean seeds contain much higher oil and fatty acid contents compared to the cereal crop seeds, including wheat, rice and maize, which results shorter viability of soybean seed in storage [17–19]. During storage, seed respiration utilizes various biomolecules viz., glucose, oils and fatty acids, which significantly reduces seed longevity, and decreases the rate of seed germination and seedling establishment, even causing soybean seeds stored for long period incapable of germination [20, 21]. The germination ability of aged soybean seed decreases considerably with increasing storage time [22]. Moreover, seed longevity is vital for the preservation of soybean genetic resources through dry seeds [23]. High germination and seedling vigor (seedling fresh weight and length) after long-term storage in both normal and low temperature conditions reduce the risk of lower yields [24]. Climate characterized by high relative humidity and temperature (humid tropical and sub-tropical climate) are the most conducive environment for seed deterioration of soybean during storage, leading to poor germination and suboptimal plant stand [25]. It has particular significance in China where the climate is very hot and humid in most part of the year, especially in the north-eastern parts which is the major soybean growing region of China [26]. Hence, development of soybean varieties that are resistant to adverse storage conditions is a most promising option to reduce loss of seed vigor and viability during storage. However, it requires the resistant genetic material, therefore soybean genotypes resistant to the adverse storage conditions must be identified. Then, genetic markers associated to seed storability should be identified to speed up breeding programmes by using marker-assisted breeding (MAB).

Quantitative trait loci (QTL) analysis is a powerful tool to decipher the molecular basis of complex traits. Variation in seed storability among soybean cultivars originating from different geographic regions have been reported [27]. Natural ageing and accelerated ageing are the two methods used in seed storability research [18, 28]. As seed storability under normal storage conditions takes years to complete, therefore artificially accelerated aging with elevated ambient temperature and relative humidity (RH) was used to rapidly assess seed storability of Arabidopsis [29], rice [12, 18], lettuce [30], wheat [31], *Brassica napus* [32] and maize [13]. Four QTLs related to seed storability of soybean on chromosomes 2, 8, 12 and 16 were identified using a F_2_ population of Birsa soya-1/ JS 71–05 [27]. Similarly, eight QTLs for seed storability were mapped on chromosome 1, 3, 4, 6, 8 and 9 of rice using doubled-haploid population that consist of 120 lines derived from the cross CJ06/TN1 [18]. Besides, ninety-six QTLs were detected for seed vigor-related traits under artificial aging condition on all wheat chromosomes except 2B, 4D, 6D, and 7D, explaining 2.9–19.4% of the phenotypic variance, using recombinant inbred line (RIL) population derived from ZB/CS [31].

In soybean, no studies for the identification of QTLs under natural aging condition have been carried out. It is very important to determine whether same or different genetic factors determine the seed storability under two different aging conditions. Although, previous studies have shown that the aging mechanism of seed under high temperature and humidity conditions is consistent with the mechanism of aging under natural conditions, but the speed of deterioration is different, and the method of artificial aging seed can be used to study the physiological and biochemical changes of natural aging seeds [33, 34]. Under natural storage conditions, QTLs controlling storability were detected in diverse genetic backgrounds of different crop species. For example, Sasaki et al. [35] identified four QTLs associated to seed longevity in rice under natural aging condition using RIL (F_7_ generation) population derived from a cross Milyang 23/Akihikari. Similarly, six QTLs linked to seed storability were identified on six chromosomes under normal aging condition in rice by using 182 backcross RILs derived from Koshihikari/Kasalath cross [36]. However, limited information is available for QTL analysis of seed storability under multiple treated conditions and diverse genetic backgrounds in soybean.

There are scarce reports related to the QTLs for soybean seed storability, and the physiological or molecular mechanisms underlying soybean seed storability are largely unclear. In the present study, the QTLs for three germination-related traits viz., GR, SL and FW under natural aging and artificial aging were detected using the two RIL population derived from a cross between the common male parent Meng 8206 with the two soybean lines viz., Zhengyanghuangdou and Linhefenqingdou. We identified the five QTL clusters/hot regions for seed storability, among which “QTL hotspot A” and “QTL hotspot B” are the major and stable genomic regions governing the inheritance of seed storability in soybean. Our results provide new insights into the genetic mechanism controlling seed storability in soybean. In addition, the QTLs reported in this study will be useful for marker-assisted breeding to achieve high seed storability.

## Results

### Correlation analysis among traits

Values of correlation coefficients among three germination-related traits based on data recorded under natural and artificial aging conditions for the LM6 and ZM6 RIL populations are presented in Table 1. The results revealed that all the three traits used to access the seed storability of soybean show significantly high positive correlation (*P* < 0.01) under both aging conditions as well as in both RIL populations (Table 1). The treatment trait value was used for correlation analysis in the natural aging, whereas relative trait value was used in case of artificial aging. This highly significant positive correlation among the three germination-related traits under both aging treatments suggests that seed storability is most likely controlled by the same genetic factors under natural and artificial conditions.

**Table 1:** Pearson’s correlation coefficients among three seed germination-related traits (GR, SL & FW) based on agronomic data of LM6 and ZM6 RIL populations under natural and artificial aging conditions

Treatment	Trait	LM6		ZM6	
		GR/rGR	SL/rSL	GR/rGR	SL/rSL
Natural aging	SL	0.552^b^		0.528^b^	
	FW	0.742^b^	0.549^b^	0.754^b^	0.567^b^
Artificial aging	rSL	0.768^b^		0.882^b^	
	rFW	0.948^b^	0.744^b^	0.884^b^	0.816^b^

In case of natural aging, the treatment trait value were used such as *GR* (germination rate), *SL* (seedling length) and *FW* (fresh weight); Under artificial aging, relative treatment value were used such as *rGR* (relative germination rate), *rSL* (relative seedling length) and *rFW* (relative fresh weight). ^a^ &^b^ represent significance at 5 and 1%, respectively

### Phenotypes of RIL mapping populations

**Fig. 1:**
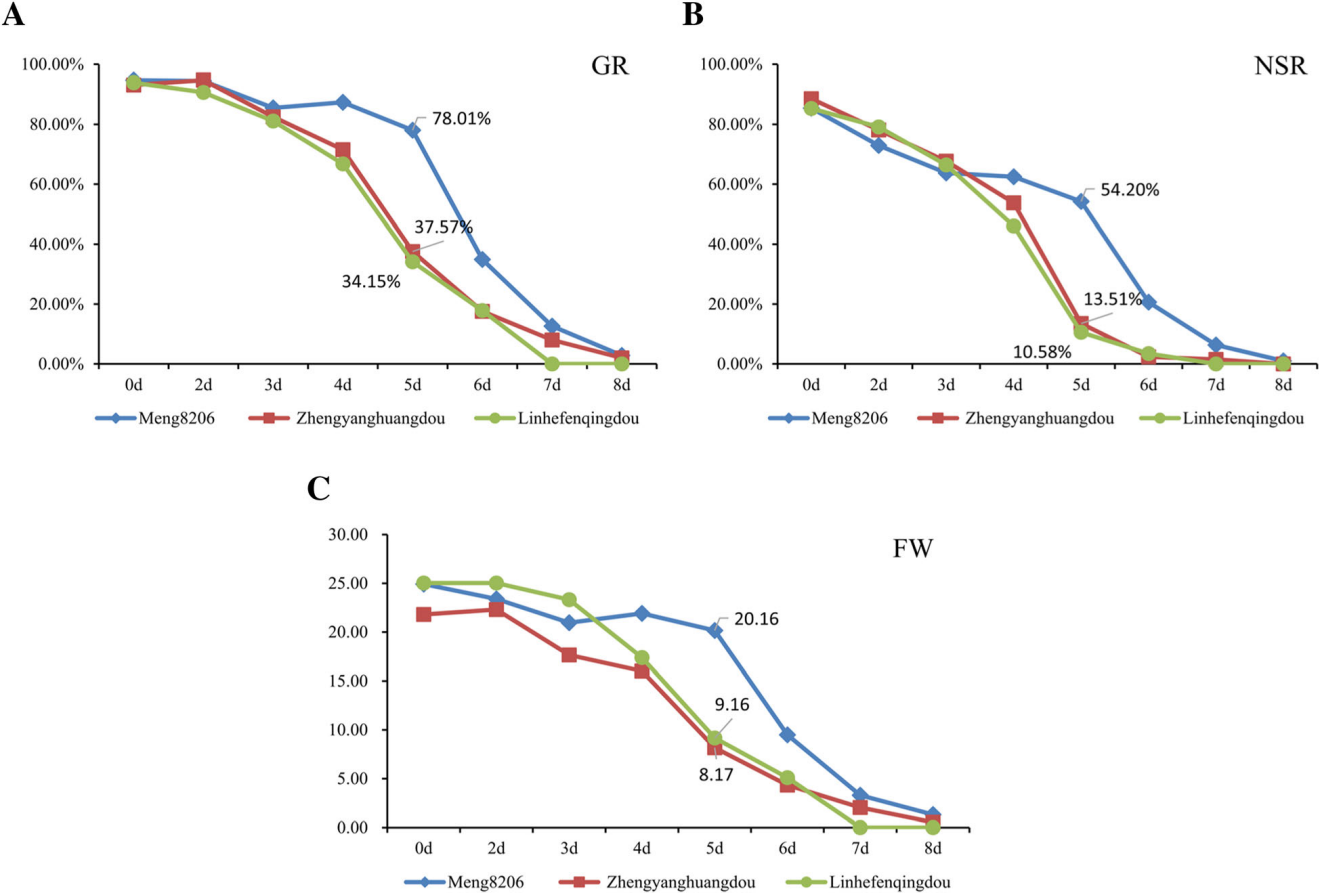
Diagram showing the performance of three parents viz., Meng8206, Zhengyanghuangdou and Linhefenqingdou of RIL populations for three traits such as germination rate (GR), normal seedling rate (NSR) and normal seedling fresh weight (FW) that are used to evaluate seed storability of soybean under artificial aging treatment. (A) Germination rate/GR; (B) Normal seedling rate/NSR; (C) Normal seedling fresh weight

Three parental accessions viz., Meng8206, Zhengyanghuangdou and Linhefenqingdou used for the construction of two RIL populations were evaluated for seed storability under artificial aging treatment by using three related traits such as GR, normal seedling rate (NSR) and FW. The Meng8206 revealed best performance for all the above three traits related to seed storability at different time intervals (0d, 1d, 3d, 4d, 5d, 6d, 7d & 8d) of artificial aging treatment compared to two female parents Zhengyanghuangdou and Linhefenqingdou (Fig. 1). This indicates higher seed storability of Meng8206 relative to female parents (Fig. 1). Descriptive statistics, ANOVA (*F*-value) and estimates of heritability showing distribution for all the three germination-related traits in both RIL populations under natural and artificial aging conditions are summarized in Table 2. The frequency distributions for the three traits viz., GR, SL and FW evaluated under natural and artificial aging for both RIL populations (LM6 & ZM6) are given in Additional file 1: Figure S1. All three traits showed normal distribution under natural aging in both RIL population, whereas these three traits displayed various levels of skewedness under artificial aging (Additional file 1: Figure S1). In case of natural aging, control was not available and only treatment value of traits were used for descriptive statistics analysis, whereas control was used for artificial aging treatment, hence both treatment and relative value have been used for this analysis (Table 2). Under natural aging treatment, the average GR was 79.60 and 79.87% for LM6 and ZM6 populations, respectively. The phenotypic values for each trait exhibited wide range, with the CV(%) ranging from 15.92% (SL) to 23.53% (FW) in LM6 population and 14.98% (SL) to 27.40% (FW) in ZM6 population. Estimates of heritability for the three traits varied from moderate (20% < H^2^ > 50%) to high (> 50%; Table 2). A minimum of 42.30% heritability was reported for SD in ZM6 population and maximum of 74.01% heritability was observed for FW in LM6 population. Furthermore, the *F*-value is highly significant for all the three traits in both RIL populations, indicating considerable phenotypic variation for these traits.

**Table 2:** Descriptive statistics (mean, SD, maximum and minimum value, CV%), heritability and *F*-value of three seed germination-related traits (GR, SL & FW) measured on LM6 and ZM6 RIL populations under natural and artificial aging treatments

Aging treatment	Traits	Mean	SD	Minimum value	Maximum value	CV (%)	Heritability(%)	*F*-value
Natural-LM6	GR (%)	79.60	12.70	38.78	100.00	15.95	53.27	2.14^**^
	SL(cm)	16.00	2.55	8.38	21.00	15.92	53.32	2.14^**^
	FW(g)	26.64	6.27	13.16	44.86	23.53	74.01	3.85^**^
Natural-ZM6	GR (%)	79.87	12.26	43.04	100.00	15.35	56.76	2.31^**^
	SL (cm)	17.51	2.62	10.43	23.51	14.98	42.30	1.66^**^
	FW (g)	21.90	6.00	5.78	33.72	27.40	64.28	2.80^**^
Artificial-LM6	GR (%)	22.40	16.31	0.00	71.60	72.83	87.34	7.90^**^
	FW(cm)	4.40	2.16	0.00	10.09	49.19	76.10	4.18^**^
	SL (g)	5.98	4.73	0.00	22.09	79.10	89.78	9.78^**^
Artificial-LM6	rGR (%)	25.62	19.24	0.00	76.20	75.09	89.13	9.20^**^
	rSL (%)	23.45	13.58	0.00	60.09	57.91	73.58	3.78^**^
	rFW (%)	16.83	14.12	0.00	66.47	83.92	88.96	9.06^**^
Artificial-ZM6	GR(%)	13.65	14.29	0.00	78.18	104.69	69.14	3.24^**^
	SL (cm)	2.50	2.24	0.00	12.36	89.58	75.49	4.08^**^
	FW (g)	2.55	3.02	0.00	16.87	118.57	75.83	4.14^**^
Artificial-ZM6	rGR (%)	14.37	16.26	0.00	90.70	113.17	83.80	6.17^**^
	rSL (%)	11.42	12.37	0.00	89.66	108.25	75.10	4.02^**^
	rFW (%)	7.87	10.43	0.00	63.93	132.48	74.63	3.94^**^

*SD* (standard deviation); *CV* (coefficient of variation); *GR* (germination rate), *SL* (seedling length) and *FW* (fresh weight); *rGR* (relative germination rate), *rSL* (relative seedling length) and *rFW* (relative fresh weight). Artificial aging includes both treatment and relative trait values, whereas natural aging includes only treatment trait value (no control was used in natural aging). * &** represent significance at 5 and 1% probability levels, respectively

In the artificial accelerated aging conditions, the relative germination rate (rGR) of LM6 and ZM6 populations were 25.62 and 14.37%, respectively. The relative phenotypic values for each trait revealed wide range, with the CV (%) ranging from 57.91% (rSL) to 83.92% (rFW) in LM6 population and 108.25% (rSL) to 132.48% (rFW) in ZM6 population. Estimates of heritability for the three traits were high (> 69%; Table 2). A minimum of 73.58% heritability was observed for rSL in LM6 population and maximum of 89.13% heritability was observed for rGR in LM6 population. Furthermore, the *F*-value is highly significant for all the three traits in both RIL populations, indicating considerable genetic variation for these traits under artificial aging condition.

Moreover, in almost all cases, the mean trait values (treatment value) in natural aging condition were higher than those in the respective artificial aging condition (Table 2). In summary, the extent of available variation and heritability for the three germination-related traits under both aging conditions were suitable for QTL analysis.

### QTL analysis for germination-related traits under natural aging condition

Genome-wide analyses were performed using the high-density genetic maps and phenotypic data of three germination-related traits (used to access seed storability) from RILs of the two populations (LM6 & ZM6) under natural aging condition. High-density genetic map of the both LM6 and ZM6 populations consist of 20 linkage groups, and contains 2267 and 2600 bin markers, respectively. The total length of the LM6 and ZM6 maps was 2453.789 cM and 2630.215 with average distance between the markers were 1.08 cM and 1.01 cM, respectively (Additional file 2: Table S1 and S2). The average length of each linkage group was 122.67 cM and 131.51 cM for LM6 and ZM6 linkage maps with the mean marker density of each linkage group was 113 and 130, respectively (Additional file 2: Table S1 and S2). In total, 16 QTLs explaining 7.82–16.76% phenotypic variation (*R*^2^) associated with three germination-related traits were detected in two RIL populations under natural aging condition (Table 3). For the LM6 population, eight QTLs associated with three germination-related traits including GR, SL and FW were identified on four chromosomes (Chr4, Chr5, Chr11 & Chr17). A single QTL explained 8.97% (*qFW-11-1*) to 15.08% (*qSL-17-2*) of phenotypic variance. Among these QTLs, three are located on Chr5 (*qGR-5-1*, *qGR-5-2* & *qFW-5-1*) and the other three are present on Chr17 (*qGR-17–1*, *qSL-17–1*& *qSL-17-2*), and the remaining two viz., *qFW-4-1* and *qFW-11-1* are located on Chr4 and Chr11, respectively (Table 3). Out of these eight QTLs, seven are major with *R*^*2*^ value > 10%, and only *qFW-11-1* is minor QTL with *R*^*2*^ value 8.97%. The most prominent QTL with the highest LOD score (5.07) was identified in a 60.91 cM region, which we designated *qGR-5-2*, explained 15.05% of phenotypic variation and displayed a negative additive effect, mainly with the positive allele from the Meng 8206. In addition, six out of eight QTLs showed negative additive effect with positive alleles from Meng 8206, only two QTLs (*qFW-4-1* and *qFW-11-1*) display positive additive effect with positive allele from Linhefenqingdou. The three QTLs detected on both Chr5 and Chr17 span approximately an interval of 13 and 17 cM, respectively on the respective chromosomes, indicating there are QTL clusters on Chr5 and Chr17. Interestingly, all the QTL within these clusters are major QTLs with *R*^*2*^ > 10% and LOD value> 3, suggesting that Chr5 and Chr17 are most likely rich in genes governing seed storability in soybean.

**Table 3:** QTLs identified for three seed-germination related traits (GR, SL & FW) in LM6 and ZM6 RIL populations under natural aging treatment

Aging treatment	Trait	QTL	Chromosome (Linkage group)	Position (cM)	Marker	LOD value	Additive effect	*R*^*2*^ value (%)
Natural-LM6	GR	*qGR-5-1*	5 (A1)	52.31	bin502-bin503	3.63	−0.05	11.13
		*qGR-5-2*	5 (A1)	60.91	bin512-bin513	5.07	−0.05	15.05
		*qGR-17–1*	17 (D2)	50.41	bin1872-bin1873	3.49	−0.06	12.98
	SL	*qSL-17–1*	17 (D2)	38.21	bin1864-bin1865	4.77	−1.04	14.53
		*qSL-17-2*	17 (D2)	45.41	bin1872-bin1873	4.44	−1.04	15.08
	FW	*qFW-4-1*	4 (C1)	92.61	bin426-bin427	3.70	2.18	10.80
		*qFW-5-1*	5 (A1)	60.91	bin512-bin513	4.46	−2.45	13.56
		*qFW-11-1*	11 (B1)	76.41	bin1201-bin1202	3.00	2.03	8.97
Natural-ZM6	GR	*qGR-5-3*	5 (A1)	71.91	bin571-bin572	6.64	0.06	16.76
		*qGR-7-1*	7 (M)	114.81	bin891-bin892	3.68	0.05	9.43
		*qGR-15–1*	15 (E)	104.01	bin1942-bin1943	3.78	−0.05	9.13
	SL	*qSL-6-1*	6 (C2)	82.61	bin681-bin682	3.20	− 0.83	8.33
		*qSL-8-1*	8 (A2)	70.51	bin958-bin959	3.04	−0.80	7.82
		*qSL-18–1*	18 (G)	96.51	bin2290-bin2290	3.19	−0.81	8.16
	FW	*qFW-5-2*	5 (A1)	71.91	bin571-bin572	3.25	1.87	8.57
		*qFW-9-1*	9 (K)	36.01	bin1118-bin1119	5.43	2.44	14.89

*GR* (germination rate), *SL* (seedling length) and FW (fresh weight); R^2^ = Coefficient of determination/phenotypic variance explained (PVE). In natural aging condition, control was not used, and we used treatment trait value for QTL analysis

In the case of ZM6 population, together eight QTLs were also identified for GR, SL and FW traits on seven chromosomes (Chr5, Chr6, Chr7, Chr8, Chr9, Chr15 & Chr18) explaining 7.82 to 16.76% of the phenotypic variance (PV) under natural aging condition (Table 3). Of the eight QTLs, six are minor with *R*^*2*^ value< 10% and only two QTLs viz., *qGR-5-3*and *qFW-9-1* are major (*R*^*2*^ value> 10%). The QTL with the highest LOD score (6.64) was identified in a 71.91 cM region, and are designated as *qGR-5-3*, explaining 16.76% of phenotypic variation, with the positive allele from the Zhengyanghuangdou (Table 3). Four QTLs (*qGR-15–1*, *qSL-6-1*, *qSL-8-1* and *qSL-18–1*) showed negative additive effect with positive allele from Meng 8206, and the remaining three QTLs viz., *qGR-7-1*, *qFW-5-2* and *qFW-9-1* displayed positive additive effects with positive alleles from Zhengyanghuangdou. The two major QTLs (*qGR-5-3*&*qFW-9-1*) identified in ZM6 population are located on Chr5 and Chr9, respectively, and in addition prominent QTLs identified in both LM6 (*qGR-5-2*) and ZM6 (*qGR-5-3*) populations are also located on Chr5, hence provides valid evidence for the important role of Chr5 in seed storability under natural aging condition.

### QTL analysis for germination-related traits under artificial aging condition

Under artificial aging condition, the relative trait value of three germination-related traits viz., rGR, rSL and rFW have been used for the mapping of QTLs for seed storability (Table 4). In LM6 population, together 10 QTLs were identified for rGR, rSL and rFW traits on five chromosomes viz., Chr3, Chr5, Chr6, Chr9 & Chr17, explaining 8.14 to 23.16% of the phenotypic variance (PV) under artificial aging condition. The four, three and three QTLs have been identified for rGR, rSL and rFW traits, respectively. Among these QTLs, four are located on Chr17 (*qrGR-17–1, qrGR-17-2, qrFW-17–1* and *qrFW-17-2*) and three are present on Chr3 (*qrGR-3-1*, *qrGR-3-2 and qrFW-3-1*), and the remaining three viz., *qrSL-5-1, qrSL-6-1* and *qrSL-9-1* are located on Chr5, Chr6 and Chr9, respectively (Table 4). Of the 10 QTLs, seven are major with *R*^*2*^ value> 10% and only three QTLs viz., *qrGR-3-*1, *qrGR-3-2* and *qrFW-3-1* have *R*^*2*^ value< 10% (minor). Most prominent QTL (*qrFW-17-2*) was identified in a 123.01 cM region having LOD score of 8.33 and *R*^*2*^ = 23.16%, with the positive allele derived from the Linhefenqingdou. Only two QTLs viz., *qrGR-3-2* and *qrSL-6-1* revealed negative additive effect with positive allele from Meng 8206, and the remaining all eight QTLs displayed positive additive effects with positive alleles from Linhefenqingdou. The four QTLs were detected in approximately 12 cM interval on Chr17 indicating there are QTL clusters on Chr17. Interestingly, all the QTLs within this clusters are major QTLs with *R*^*2*^ > 10% and LOD value> 6, this further corroborate that Chr17 is most likely rich in key genes controlling seed storability in soybean.

**Table 4:** QTLs identified for three seed-germination related traits (GR, SL & FW) in LM6 and ZM6 RIL populations under artificial aging treatment

Aging treatment	Traits	QTL	Chromosome (Linkage group)	Position(cM)	Marker	LOD value	Additive effect	*R*^*2*^ value(%)
Artificial-LM6	rGR	*qrGR-3-1*	3 (N)	1.01	bin228-bin229	3.37	0.06	8.94
		*qrGR-3-2*	3 (N)	81.01	bin322-bin323	3.43	−0.05	8.14
		*qrGR-17–1*	17 (D2)	113.31	bin1924-bin1925	7.05	0.09	19.11
		*qrGR-17-2*	17 (D2)	123.01	bin1930-bin1931	8.65	0.09	22.64
	rSL	*qrSL-5-1*	5 (A1)	55.51	bin505-bin506	4.09	0.01	11.78
		*qrSL-6-1*	6 (C2)	62.11	bin613-bin614	4.06	−0.01	11.75
		*qrSL-9-1*	9 (K)	77.51	bin1057-bin1058	4.12	0.02	11.92
	rFW	*qrFW-3-1*	3 (N)	3.31	bin231-bin232	3.50	0.04	8.51
		*qrFW-17–1*	17 (D2)	116.51	bin1925-bin1926	6.41	0.07	19.05
		*qrFW-17-2*	17 (D2)	123.01	bin1930-bin1931	8.33	0.07	23.16
Artificial-ZM6	rGR	*qrGR-10-1*	10 (O)	91.21	bin1313-bin1314	3.73	0.05	8.69
		*qrGR-17-3*	17 (D2)	130.21	bin2176-bin2177	4.26	−0.05	10.56
		*qrGR-18–1*	18 (G)	47.91	bin2226-bin2227	6.61	0.07	16.03
	rSL	*qrSL-17–1*	17 (D2)	128.21	bin2176-bin2177	3.09	−0.03	7.38
		*qrSL-18–1*	18 (G)	35.21	bin2218-bin2219	3.05	0.03	7.38
		*qrSL-18-2*	18 (G)	50.01	bin2228-bin2229	3.01	0.03	7.30
	rFW	*qrFW-11-1*	11 (B1)	3.51	bin1356-bin1357	3.25	−0.04	8.50
		*qrFW-17-3*	17 (D2)	129.21	bin2176-bin2177	4.26	−0.04	11.44

*rGR* (relative germination rate), *rSL* (relative seedling length) and *rFW* (relative fresh weight); R^2^ = Coefficient of determination/phenotypic variance explained (PVE). In artificial aging condition control was available, and we used relative trait value for QTL analysis

For the ZM6 population, eight QTLs were identified for rGR, rSL and rFW traits on four chromosomes (Chr10, Chr11, Chr17 & Chr18) under artificial aging (Table 4). A single QTL explained 7.30% (*qrSL-18-2*)-16.03% (*qrGR-18–1*) of phenotypic variance. Out of these eight QTLs, five are minor with *R*^*2*^ value < 10%, and the remaining three viz., *qrGR-17-3*, *qrGR-18–1* and *qrFW-17–1* are major QTLs with *R*^*2*^ value> 10. The *qGR-18–1* was identified as the most prominent QTL with the highest LOD score (6.61), explained 16.03% of phenotypic variation and displayed a positive additive effect, with the positive allele from the Zhengyanghuangdou. In addition, four QTLs viz., *qrGR-17-3*, *qrSL-17–1*, *qrFW-11-1* and *qrFW-17-3* showed negative additive effect with positive alleles from Meng 8206, and the remaining three QTLs display positive additive effect with positive allele from Zhengyanghuangdou (Table 4). The three QTLs were detected in approximately 3 cM interval on Chr17 indicating there are QTL clusters on Chr17. Interestingly, two out of three QTLs within this cluster are major QTLs with *R*^*2*^ > 10% and LOD value> 4. Hence, it is confirmed from both aging conditions and RIL populations, that Chr17 plays important role in governing inheritance of seed storability in soybean (Tables 3 & 4).

### “QTL hotspots” and stable genomic regions for seed storability

**Fig. 2:**
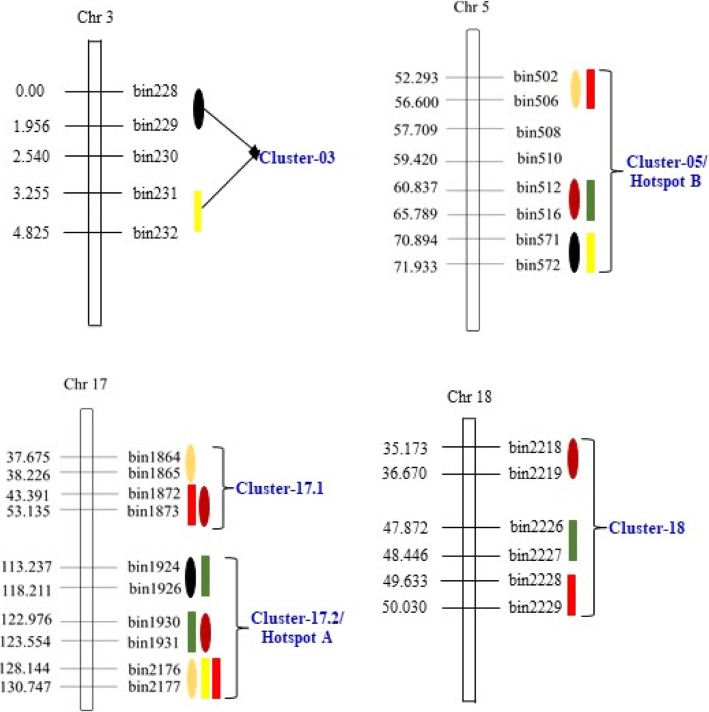
Diagram showing the location of five QTL clusters/hot regions (cluster-03, cluster-05, cluster-17.1, cluster-17.2 and cluster-18) on four different chromosomes viz., Chr3, Chr5, Chr17 and Chr18 identified in LM6 and ZM6 RIL populations under natural and artificial aging conditions

QTL cluster/hotspots is defined as a densely populated QTL region of the chromosome that contains multiple QTLs associated with various traits. In this study, we found five QTL clusters in four different chromosomes viz., Chr3, Chr5, Chr17 &Chr18, and were named as Cluster-03, Cluster-05, Cluster-17.1, Cluster-17.2 and Cluster-18, respectively (Table 5). The highest concentration of QTLs for seed storability was identified in Cluster-17.2 of Chr17, and is designated as “QTL hotspot A” spanning physical length of 1.4 Mb (Fig. 2). This QTL hotspot harbors seven QTLs (six major and one minor) viz., *qrGR-17–1*, *qrGR-17-2*, *qrFW-17–1*, *qrFW-17-2*, *qrGR-17-3*, *qrSL-17–1* and *qrFW-17-3* associated to all three studied germination-related traits (Table 5). All the QTLs underlying “QTL hotspot A” are major QTLs (R^2^ > 10%) expect *qrSL-17–1*, and explained 7.38–23.16% of phenotypic variation (Table 5). Another sets of QTL rich region was found in Cluster-05 on Chr5, and is designated as “QTL hotspot B” with length of 4.0 Mb. It harbors six QTLs for all three germination-related traits that includes *qGR-5-1*, *qGR-5-2*, *qFW-5-1*, *qGR-5-3*, *qFW-5-2* and *qrSL-5-1*. Out of these six QTLs, five are major (*R*^*2*^ > 10%) with only *qFW-5-2* as minor QTL (*R*^*2*^ < 10%), and are explaining 8.57–16.76% of phenotypic variation (Table 5). The Cluster-3 contain two QTLs for two traits viz. rGR and rFW, explaining phenotypic variation of 8.51–8.94%, whereas Cluster-17.1 and Cluster-18 contain three QTLs each, explaining phenotypic variation of 12.98–15.08% and 7.30–16.03%, respectively (Table 5). Furthermore, the QTLs within “QTL hotspot B” have been identified in both RIL populations as well as aging conditions, and in addition QTLs underlying “QTL hotspot A” have been identified in both RIL populations under artificial aging condition (Fig. 2**,** Table 5). Hence, these are the stable genomic regions governing the inheritance of seed storability in soybean.

**Table 5:** Five QTL hotspots/clusters detected in LM6 and ZM6 RIL population under natural and artificial conditions

QTL cluster name	Chr_Bin range	Physical range (bp)	QTL Name	LOD	Additive effect	*R*^2^ (%)
Cluster-03	Chr03_bin228-bin232 (LM6)	00000001–00533669	*qrGR-3-1*	3.37	0.06	8.94
			*qrFW-3-1*	3.5	0.04	8.51
Cluster-05/ QTL hotspot B	Chr05_bin502-bin513 (LM6) Chr05_bin571-bin572 (ZM6)	31,127,720–35,178,169	*qGR-5-1*	3.63	−0.05	11.13
			*qGR-5-2*	5.07	−0.05	15.05
			*qFW-5-1*	4.46	−2.45	13.56
			*qGR-5-3*	6.64	0.06	16.76
			*qFW-5-2*	3.25	1.87	8.57
			*qrSL-5-1*	4.09	0.01	11.78
Cluster-17.1	Chr17_bin1864-bin1873 (LM6)	77,540,160–10,193,909	*qGR-17–1*	3.49	−0.06	12.98
			*qSL-17–1*	4.77	−1.04	14.53
			*qSL-17-2*	4.44	−1.04	15.08
Cluster-17.2/ QTL hotspot A	Chr17_bin1924-bin1931 (LM6) Chr17_bin2176-bin2177 (ZM6)	39,676,735–41,073,260	*qrGR-17–1*	7.05	0.09	19.11
			*qrGR-17-2*	8.65	0.09	22.64
			*qrFW-17–1*	6.41	0.07	19.05
			*qrFW-17-2*	8.33	0.07	23.16
			*qrGR-17-3*	4.26	−0.05	10.56
			*qrSL-17–1*	3.09	−0.03	7.38
			*qrFW-17-2*	4.26	−0.04	11.44
Cluster-18	Chr18_bin2218-bin2229 (ZM6)	05407986–07447039	*qrGR-18–1*	6.61	0.07	16.03
			*qrSL-18–1*	3.05	0.03	7.38
			*qrSL-18-2*	3.01	0.03	7.30

All the model genes within the physical intervals of “QTL hotspot A” and “QTL hotspot B”, and their gene annotations were downloaded from the Phytozome (https://phytozome.jgi.doe.gov) and Soybase (http://www.soybase.org) databases. A total of 159 and 455 gene models were found in the “QTL hotspot A” and “QTL hotspot B” respectively. After screening 19 genes showed relationship with seed development, seed germination, seed dormancy, seed coat formation, fatty acid/lipid metabolic process and seed storage (Additional file 2: Table S3). Hence, based on their function we considered them as a possible candidate for seed storability. However, it needs further validation to prove their actual role in soybean seed storability.

## Discussion

Poor storability results in considerable production and economic losses due to the impossibility of carry-over of seed lots, which have lost their vigor and viability, and are no longer marketable. Soybean seeds decline in quality very faster relative to the seeds of other crops [37]. It is a major problem for soybean production in the tropics and sub-tropics, where rapid loss of seed germination capacity occurs during storage under ambient storage conditions [38]. Moreover, in majority of countries especially developing and under-developed, most farmers have poor seed processing and storage facilities, resulting the viability of seed to lose faster. Storability is an important agronomic trait as far as crop production and germplasm conservation in gene banks is concerned [24, 39, 40]. Poor storability leads to unexpected losses in seed viability during storage and negatively impacts seedling establishment, crop production and yield. Hence, improving seed storability is important to increase overall crop production as well as play key role to maintain the global food security [15]. It is a complex quantitative trait, and is considerable influenced by growing environments. Despite the substantial research on storability, its mechanism is not clear and breeders have not applied currently available information. However, to breed for storability resistance, a full understanding of the genetic basis of resistance is indispensable. In this study, three different germination-related parameters were used for evaluating seed storability resistance under two different aging treatments (natural and artificial), and each trait revealed moderate to high broad-sense heritability. This indicates that storability resistance is environmentally stable in our study and mainly controlled by genetic factors. The high correlation coefficients of germination-related parameters under both natural and artificial aging conditions supported this result. Particularly, the highly significant correlation coefficient of GR with FW and SL implies that high GR is correlated with high FW and SL.

QTL mapping has been commonly used for the QTL/gene identification in crop plants, and is an efficient approach to analyze quantitative traits. The quality of genetic maps has a great influence on the accuracy of QTL detection, and therefore increasing marker density can increase the resolution of genetic maps for a given mapping population [41, 42]. Hence, it is prerequisite to prepare high-density linkage maps and thereby improve the efficiency and accuracy of linkage mapping and MAS. In this study, we used two high-density genetic maps of the LM6 and ZM6 populations contains 2267 and 2600 bin markers, respectively. The markers in both linkage maps were integrated to all the 20 LGs, and the average distance between adjacent markers was only 1.08 cM and 1.01 cM in case of LM6 and ZM6, respectively. These high-density genetic maps could ensure that a molecular marker and QTL were tightly linked, and provided a good foundation for analyzing quantitative traits.

Soybean seeds in storage or gene bank preservation of natural conditions gradually lose their germination ability. Natural aging lasts long time usually more than one year, and is difficult to apply for germplasm screening. Instead, artificial aging uses high temperature and humidity to model and speed the natural aging, and has been widely used in seed processing research [30, 43, 44]. However, only limited QTL studies have been carried out for seed longevity in soybean that have identified few QTLs associated with seed longevity and seedling vigor [27]. These QTLs were located on chromosomes 2, 8, 12 &16. Moreover, most of the research on longevity was based on accelerated ageing [45]. However, results from natural and accelerated ageing indicate that there may be genetic differences in the results from the two systems [46]. We therefore used both natural and artificial aging conditions to elucidate whether or not the artificial aging mimic with natural aging completely. In this study, *qGR-5-1* and *qrSL-5-1* have been reported on Chr5 of LM6 population between the same bin502-bin506 marker interval under both natural and artificial aging treatment, respectively. The rest of the QTLs identified for seed storability under natural aging condition do not fall in a common marker-interval of the QTLs identified in artificial aging condition. This suggests that artificial aging treatments do not completely mimic deterioration process in conventional storage conditions. Some previous studies reported different deterioration mechanisms might be involved in natural and artificial accelerated storage [30]. Conversely, Likhatchev et al. [47] revealed that physiological changes in seeds were same under both conventional and artificial aging conditions. Similarly, by comparing the biochemical behavior of seeds under natural and artificial aging, it was suggested that similar molecular events occurs at both aging conditions, and artificial aging mimics the natural seed aging as was indicated by germination behavior [48]. The identification of common QTL (*qGR-5-1* and *qrSL-5-1*) between the same marker interval under both aging condition in this study seems to support the latter conclusion. However, except *qGR-5-1* and *qrSL-5-1*, the remaining QTLs identified in artificial aging treatment are not located at same marker interval of QTLs reported in natural aging, hence indicating that artificial aging does not completely mimic deterioration process in conventional storage conditions.

In the present study, a total of 16 and 18 QTLs were identified for seed storability under natural and artificial aging conditions, respectively. The nine (*qGR-5-1*, *qGR-5-2*, *qGR-17–1*, *qSL-17–1*, *qSL-17-2*, *qFW-4-1, qFW-5-1*, *qGR-5-3*& *qFW-9-1*) and ten (*qrGR-17–1*, *qrGR-17-2*, *qrSL-5-1*, *qrSL-6-1*, *qrSL-9-1*, *qrFW-17–1*, *qrFW-17-2*, *qrGR-17-3*, *qrGR-18–1* & *qrFW-17-3*) QTLs could be considered as the major QTLs for seed storability under natural and artificial aging condition, respectively, because of their larger LOD values (> 3.5) and explained more of the PV(> 10%). Because some QTLs associated with seed storability/longevity have been previously reported, it is very important to identify novel QTLs associated with seed storability. Based on the QTLs listed in SoyBase (www.soybase.org), all the QTLs identified in this study are novel. Previously, the QTLs for seed longevity have been identified on four chromosomes viz., 2, 8, 12 & 18 [27]. But in the present study the QTLs were identified on different chromosomes viz., [3–9, 11, 15, 17, 18], although one QTL (*qSL-8-1*) has been identified on Chromosome 8 but that lies at considerable distance from the one identified previously (seed viability 1–1) associated with Satt538 marker [27]. Moreover, all the seed longevity QTLs identified earlier were minor explaining less than 7.7% phenotypic variation (*R*^*2*^ < 0.075), and these QTLs have not been further characterized, validated as well as used in marker-assisted breeding for the improvement of seed storability in soybean. One of the reason will be the use of low-density linkage map based on low-throughput markers (SSR) in these studies, which does not provide the required information (i.e., number and resolution of QTLs/genes) as needed for marker-assisted breeding. Based on the high-density genetic map, the confidence interval for most of the QTLs was less than 5 cM, and each QTL had two or more closely linked markers (within 0–5 cM). These loci are favorable for the MAS of QTLs by soybean breeding programs. Therefore, there was considerable lack of information about the genetic architecture of seed storability in soybean. Hence, the present study provides detailed understanding about QTLs/genes governing the inheritance of seed storability in soybean, and added information to the growing knowledge on the genetic control of seed storability.

Co-localization of QTL on chromosomes were detected for three germination-related traits determining the degree of seed storability in this study. QTL co-localization on chromosomes, referred to as “QTL cluster/hotspots”, have been previously reported in soybean [49, 50]. In the present research, a few genomic regions containing QTL clusters were examined, and five QTL clusters were found on four chromosomes viz.,3, 5, 17 and 18 (Fig. 2). These QTL clusters affected two or more different seed germination-related traits. The highest number of seven and six QTLs were observed on “QTL hotspot A” and “QTL hotspot B”, respectively harboring QTLs for all the three seed-germination related traits viz., GR, FW and SL (Fig. 2**,** Table 5). The other three clusters viz., Cluster-3, Cluster-17.1 and Cluster-18 contain two, three and three QTLs, respectively for at least two germination-related traits (Table 5). These QTLs clusters have not been published and add to the growing knowledge on the genetic control of these traits. The phenomenon of QTL clustering might represent the linkage of genes and QTL or result from pleiotropic effects of a single QTL in the same genomic region [49]. This co-localization of QTLs for three different germination-related traits was in accordance with the fact that all of them were highly significantly correlated with each other (Table 1). These QTL hotspot regions revealed that the linkage/pleiotropy QTLs will facilitate the improvement of seed storability. Previously, some of the QTLs for other traits have been also identified in the same region of “QTL hotspot A” on chromosome 17, that are related to oil content and fatty acid composition of soybean seed [51], days to flowering and maturity [52]. Similarly, earlier studies have also reported QTLs for seed oil, seed protein, flowering, seed set, shoot Fe & Mg content and ureide content in the “QTL hotspot B” region on Chr5 [52–56]. The defective seed maturation leads to rapid loss of viability upon storage as has been shown for *leafy cotyledon1* (*lec1*) and *abscisic acid intensitive3* (*abi3*) mutants [57–59]. Seed maturity, viability and storability are correlated with each other [60]. The oil content and fatty acid composition plays an essential role in the viability of soybean seed during storage conditions. Seeds rich in lipids have poor longevity due to their specific chemical composition [61]. For example, soybean seed storage demands special attention due to high oil content, otherwise, processes may occur that lead to loss of germination ability and seed viability [61]. The development of rancidity has been recognized as the predominant cause of oil deterioration and reduction during storage [62]. This suggesting the potential probability of common genic factors for these traits, and also showing the necessity to promote further study for these regions. These QTL clusters have provided some valuable information to define genome regions with different traits. Based on the comprehensive analysis of clusters in this study, breeding programs targeting increase of seed storability with superior oil quality can focus on hotspot clustering areas and select around the region. Lastly, existence of QTL clusters/hotspots has provided proof that genes related to some crop traits are more densely concentrated in certain genomic regions of crop genomes than others [63, 64].

## Conclusion

In conclusion, the present study is the first detailed and comprehensive investigation of QTLs for seed storability in soybean, in which we used high-density linkage maps of two RIL populations (LM6 and ZM6) for the discovery of storability QTLs. We identified 34 QTLs for three seed germination-related traits (used to access seed storability), out of which 19 are major QTLs with *R*^*2*^ > 10% and LOD > 3. All the identified QTLs are novel, and has not been previously reported in soybean. Furthermore, 21 identified QTLs are clustered to five QTL clusters, among which “QTL hotspot A” and “QTL hotspot B” are the major and stable genomic regions governing the inheritance of seed storability in soybean. These regions could be the main focus of soybean breeders for fine mapping, candidate gene identification as well as marker-assisted selection of soybean genotypes with superior seed storability. Lastly, the improvement of seed storability in soybean will play considerable role in increasing overall production and yield, as well as avoid losses that results due to poor seed storability. Hence, seed storability has great importance in maintaining sustainable crop production as well as global food security.

## Methods

### Plant materials

In the present study, we used two sets of recombinant inbred line populations (RILs) developed from the Zhengyanghuangdou × Meng 8206 (ZM6) and Linhefenqingdou × Meng 8206 (LM6) crosses that consist of 126 and 104 lines, respectively. The Meng8206 parent (male) has good seed longevity, whereas Zhengyanghuangdou and Linhefenqingdou (female parents) have low seed longevity (Fig. 1). All the three parental accessions viz., Meng8206, Zhengyanghuangdou and Linhefenqingdou have been received from Soybean Germplasm Gene Bank at National Center for Soybean Improvement (Ministry of Agriculture), Nanjing Agricultural University, Nanjing, China, that are deposited there with Accession ID of N21257, N05082 and N06141, respectively. The average flowering/maturity time for Meng8206, Zhengyanghuangdou and Linhefenqingdou were 39.3/105, 42.5/108 and 49.9/115, respectively. Parental accessions and RIL populations were both planted in a randomized complete block design with three replications in the field at Jiangpu agricultural experiment station of Nanjing Agricultural University in the growing seasons of 2011 and 2014. The harvested seeds of both the populations were sundried until about 10% seed moisture, and then were used for evaluation of seed storability.

### Natural and artificial aging treatment

#### Natural aging

The seeds from both the RIL population planted in 2011 growing season were harvested in November 2011, and stored in the Seed Storage Room of National Soybean Improvement Center, Nanjing Agricultural University till July 2015. During this storage period the temperature was maintained at 20 °C in the months of April–October but at room temperature in the remaining months i.e., November–March, with no control of humidity (40–70%). In July 2015, seeds were removed from storage room for storability test [65].

#### Artificial accelerated aging

The artificial aging (AA) experiment was performed to determine the optimal aging conditions and duration [66]. Accelerated aging method of Singh and Ram [25], who kept seeds at 40 °C and 100% RH (relative humidity) for 96 h, was modified in an attempt to ensure clear-cut differences between seeds of superior and poor storability. The 100% RH required for the AA test method as specified in the International Seed Inspection Code (ISIC) is difficult to achieve, and the seed was extremely sensitive at 100% RH. Fresh seed samples harvested in 2014 growing season of both RIL populations were enclosed in small nylon bags that are kept orderly in the aging box for 5 days under the artificial accelerated aging conditions of 45 °C ± 1 and 95 ± 1% RH. Seeds were subjected to standard germination tests randomly. The seed aging box was provided by Shanghai Qi Xin Scientific Instrument Co., Ltd., model LH-150S [65].

### Evaluation of seed storability

In accordance to GB / T3543.4–1995 “*Rules for agricultural seed testing procedure*”, the standard technical requirements for soybean germination was applied (https://www.chinesestandard.net/PDF/English.aspx/GBT3543.4-1995). Fifty seeds with intact seed coat of each line exposed to either natural or artificial ageing conditions in two replicates were used for storability test. For germination test, the seeds were initially disinfected with 70% ethanol solution, then washed three times with sterile water and dried with a paper roll method [67], and dipped in a turnover box containing distilled water to a height of about 3 cm, under the conditions of room temperature (25 °C) for 5 days to ensure normal germination.

For the evaluation of seed storability, the following three parameters related to seed germination and vigor were used viz., germination rate (GR; Number of germinated seeds/Total number of seeds × 100), normal seedling length (SL) and normal seedling fresh weight (FW). In case of artificial aging, the control was also used that does not experience any aging treatment, hence the relative trait value were used for analysis such as relative germination rate (rGR), relative normal seedling length (rSL) and relative normal seedling fresh weight (rFW). The relative value of these three traits were calculated by the formula as Trait Value under Aging Treatment/Trait Value of control × 100 (%). However, in case of natural aging no control was available, therefore we used treatment trait value for analysis [65].

### SNP genotyping and bin map construction

Map construction begins with the extraction of genomic DNA from the young leaves of two RIL populations following the protocol of Zhang et al. (2004) [68]. This genomic DNA was digested using *Taq I* to construct genomic DNA library following Baird et al. [69]. DNA fragments between 400 and 700 bp were selected as well as sequenced using the Illumina HiSeq 2000 standard protocol for MSG (multiplexed shotgun genotyping), and 90-mer paired-end reads were generated [70]. SOAP2 software was used for aligning the sequenced reads to the Williams 82 reference genome [71]. SNP calling and genotyping were conducted using Real SFS software [72], based on the Bayesian estimation. Subsequently, using a three-standard filter, 50 < depth < 2500, a probability of site mutation 95%, and every SNP loci separated by at least 5 bp, we obtained high confidence SNPs.

Bin maps were constructed using a sliding window approach. The SNPs were used as genomic markers, from which another type of genomic marker, i.e., bin marker were derived. The genomic bin markers were constructed using a slightly modified sliding-window approach developed by Huang et al. [73] based on the SNP dataset without imputation. Consecutive SNPs were scanned with a window size of 15 SNPs and a step size of one SNP. Windows with 11 or more SNPs from either parent were considered to be homozygous but those with fewer SNPs from a single parent were considered heterozygous. Recombination breakpoints were determined by the SNP position that switched one genotype to another consecutive genotype. Consecutive intervals of 30-kb that do not possess any recombination event within the population were combined into bins, and these bins were used as markers. According to the breakpoint information, the bin information was generated using a PERL script [74]. The linkage maps of bin markers were constructed for each of the two RIL populations using JoinMap 4.0 [75].

### Data analysis and QTL mapping analysis

Descriptive statistics like mean, standard deviation (SD), maximum and minimum trait value, coefficient of variation (CV%), analysis of variance (ANOVA) and heritability for each seed germination-related trait, and correlations among pairs of traits were calculated using the SPSS17.0 software (http://www.spss.com).

The QTL analysis was performed on WinQTLCart2.5 software with the model of composite interval mapping [76]. A 10-cM window at a walking speed of 1 cM was used in a stepwise forward regression procedure. The LOD threshold was calculated using 1000 permutations for an experimental-wise error rate of *P =* 0.05. The additive effects (Add) and phenotypic variance explained by QTL (*R*^2^) were estimated according to the bin at the highest peaks as determined by WinQTLCart2.5. The QTL was named following normal nomenclature [77].

### Candidate gene prediction

Soybean genomic data from the physical interval position of “QTL hotspot A” and “QTL hotspot B” were downloaded from the SoyBae (http://www.soybase.org) and Phytozome (http://phytozome.jgi.doe.gov) database, and candidate genes were predicted based on the gene annotations information (http://www.soybase.org; https://phytozome.jgi.doe.gov). Candidate genes were screened by using the gene ontology (GO) information from SoyBase through online resources: GeneMania (http://genemania.org/), Gramene (http://archive.gramene.org/db/ontology), Kyoto Encyclopedia of Genes and Genomes website (KEGG, www.kegg.jp) and National Center for Biotechnology Information (NCBI: https://www.ncbi.nlm.nih.gov).

## Additional files

Additional file 1: **Figure S1.** Frequency distribution of germination rate (GR), normal seedling length (SL) and normal seedling fresh weight (FW) of LM6 and ZM6 RIL populations under natural and artificial aging conditions. For natural aging only treatment trait were used for analysis, whereas for artificial aging both treatment and relative trait value have been used for analysis. Relative germination rate (rGR); Relative normal seedling length (rSL); Relative normal seedling fresh weight (rFW). (DOCX 173 kb)

Additional file 2: **Table S1.** Distribution of SNPs, recombination bins and markers mapped on soybean chromosomes/linkage groups of LM6 RIL population. Bin-map (RAD-sequencing). **Table S2.** Distribution of SNPs, recombination bins and markers mapped on soybean chromosomes/linkage groups of ZM6 RIL population. Bin-map (RAD-sequencing). **Table S3.** Possible candidate genes predicted within “QTL hotspot A” and “QTL hotspot B” regions for soybean seed storability based on known functional annotations (XLSX 21 kb)

## Data Availability

The data sets supporting the results of this study are included in the manuscript. Soybean seeds are available from the National Center for Soybean Improvement, Nanjing Agricultural University, PR China.

## Abbreviations

(rFW): Relative normal seedling fresh weight
(rGR): Relative germination rate
(rSL): Relative normal seedling length
AA: Artificial aging
ANOVA: Analysis of variance
Chr: Chromosome
CV: Coefficient of variation
FW: Normal seedling fresh weight
GR: Germination rate
MAB: Marker-assisted breeding
MSG: Multiplexed shotgun genotyping
PV: Phenotypic variance
QTL: Quantitative trait loci
*R*^*2*^: Coefficient of determination
RH: Relative humidity
RIL: Recombinant inbred line
SD: Standard deviation
SL: Normal seedling length
SNP: Single nucleotide polymorphism
