# Supplementary material for: Whole-genome mapping identified novel “QTL hotspots regions” for seed storability in soybean (Glycine max L.)
Source: BMC Genomics. 2019 Jun 17;20:499. doi: 10.1186/s12864-019-5897-5 (PMC6580613; doi:10.1186/s12864-019-5897-5)
Supplement: Supplementary file 1 — Figure S1. Frequency distribution of germination rate (GR), normal seedling length (SL) and normal seedling fresh weight (FW) of LM6 and ZM6 RIL populations under natural and artificial aging conditions. For natural aging only treatment trait were used for analysis, whereas for artificial aging both treatment and relative trait value have been used for analysis. Relative germination rate (rGR); Relative normal seedling length (rSL); Relative normal seedling fresh weight (rFW). (DOCX 173 kb) [file 12864_2019_5897_MOESM1_ESM.docx]

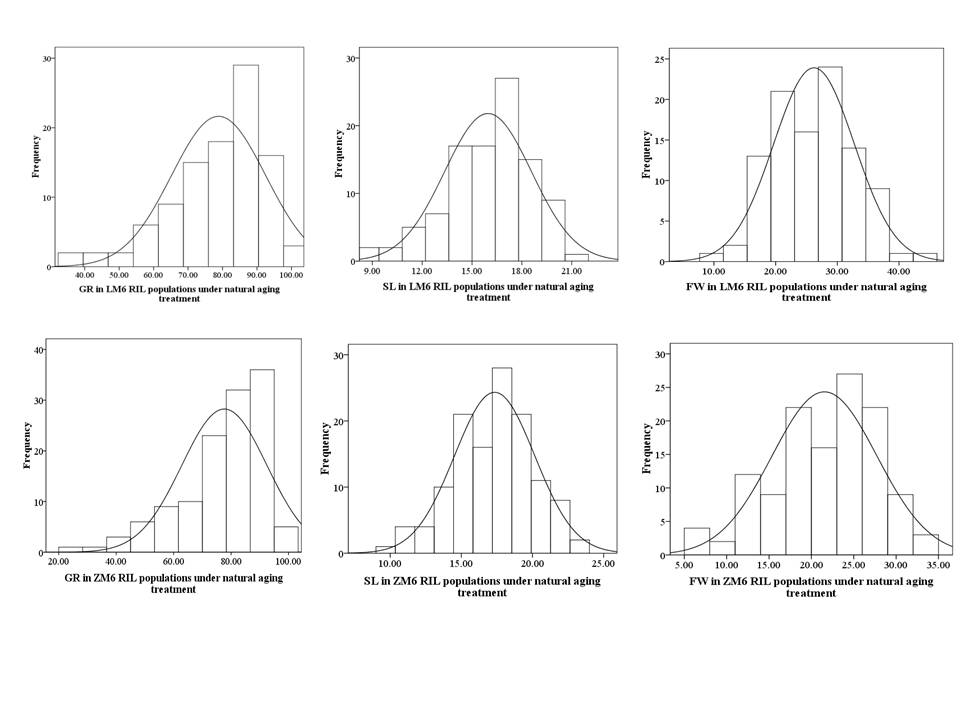


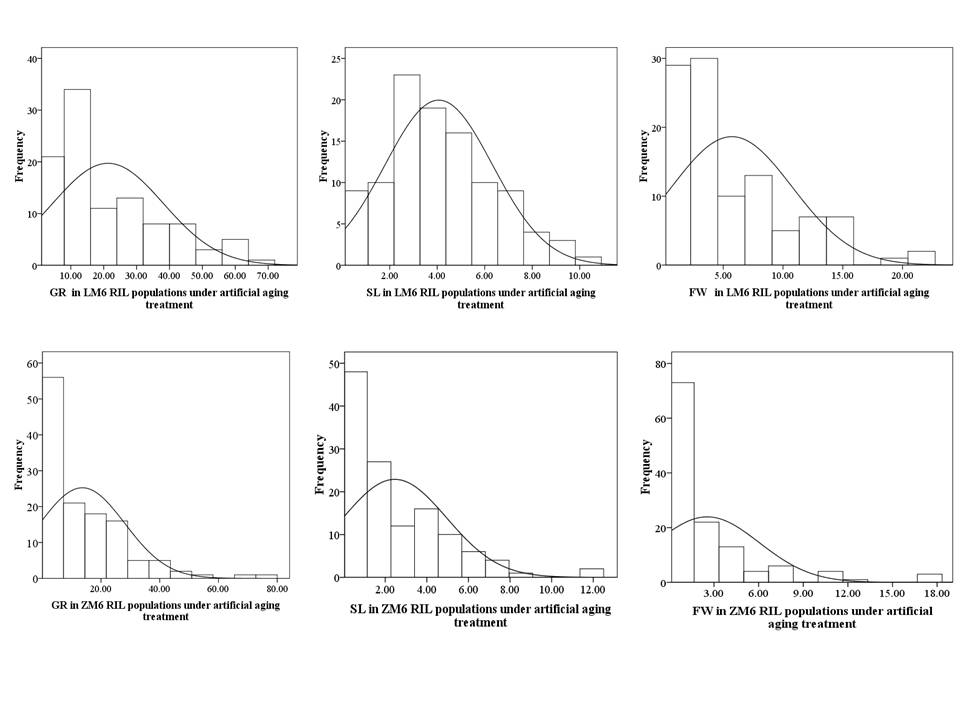


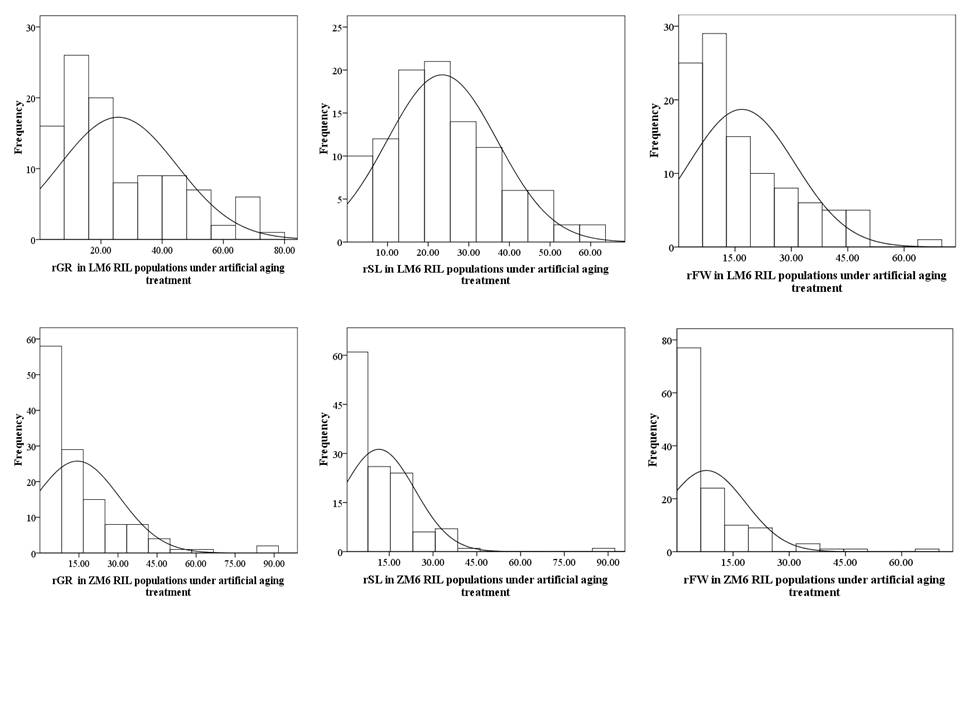


**Supplementary Fig. S1:** Frequency distribution of germination rate (GR), normal seedling length (SL) and normal seedling fresh weight (FW) of LM6 and ZM6 RIL populations under natural and artificial aging conditions.

For natural aging only treatment trait were used for analysis, whereas for artificial aging both treatment and relative trait value have been used for analysis. **Relative germination rate (rGR); Relative normal seedling length (rSL); Relative normal seedling fresh weight (rFW).**
